# Holding the Line – Mental Well-Being, Stressors, and Coping in Crisis Supporters

**DOI:** 10.1027/0227-5910/a000985

**Published:** 2024-12-11

**Authors:** Jayden Sercombe, Emma K. Devine, Mark Deady, Katherine L. Mills

**Affiliations:** ^1^ The Matilda Centre for Research in Mental Health and Substance Use, The University of Sydney, NSW, Australia; ^2^ Faculty of Medicine and Health, The Black Dog Institute, The University of New South Wales, Sydney, NSW, Australia

**Keywords:** crisis helpline, crisis supporters, compassion fatigue, mental well-being, occupational mental health

## Abstract

**Abstract:** *Background:* Crisis supporters can experience numerous stressors in their role that can impact their own mental well-being. The area remains underexplored in research, particularly relating to substance use, and new trends in the role such as working remotely or the impact of providing chat-based support. *Aims:* This study identifies crisis support-related stressors, as well as levels of mental well-being and substance use, and factors associated with mental well-being. *Method:* Participants (*n* = 422) were recruited from four leading crisis support services and via social media advertising. They responded to an online survey, assessing demographics, stressors, mental well-being (compassion fatigue, compassion satisfaction, and psychological distress), substance use, and coping styles. *Results:* Findings identified several important stressors (e.g., argumentative callers) and moderate to high rates of compassion fatigue and psychological distress. High levels of compassion satisfaction were reported, and levels of risky substance use were low. Problem-focused coping emerged as a key factor related to positive mental well-being, while emotion-focused, avoidant coping, remote work, and providing chat-based support were linked to negative well-being. *Limitations:* The study’s cross-sectional design and convenience sample limit causal inferences and generalizability. *Conclusion:* The findings reveal significant stressors and challenges in crisis supporters that require consideration and intervention.

Crisis helplines play a vital role in suicide prevention globally (The World Health Organisation [WHO], 2013). Crisis-focused counseling delivered via helplines has demonstrated effectiveness in reducing caller suicidal ideation and intent (Gould et al., 2007; Williams et al., 2021). Crisis helplines rely on the dedicated work of crisis supporters, who provide support over phone, online chat, or mobile text to people experiencing loneliness, suicidality, emotional distress, and other mental health challenges (Roche & Ogden, 2017). In this role, supporters are exposed to vivid descriptions of traumatic material, suicidality, as well as complex caller profiles, including *frequent callers* (who may call many times a day) and *sex callers* (who exploit the line for sexual gratification; Pirkis et al., 2016; Willems et al., 2020). Adding to these stressors, due to the one-off nature of contact to crisis lines, crisis supporters are unable to anticipate the trajectory or content of a call before answering it (Kitchingman et al., 2024).

The demanding nature of the work can impact the mental well-being of crisis supporters (Kitchingman, Wilson, et al., 2018). Over three in four Australian crisis supporters exhibit symptoms of compassion fatigue, defined as stress resulting from empathetic engagement with others and their trauma (Bride et al., 2007; O’Sullivan & Whelan, 2011). Crisis supporters also report elevated psychological distress (e.g., Kitchingman et al., 2017), which is predictive of mental health disorders (Sunderland et al., 2011). Other helping professionals have reported harmful substance use, perhaps as a way of coping with their role-related stressors (Bonumwezi et al., 2022), but it remains unclear whether this is also the case among crisis supporters.

Crisis support workers are differentially impacted by the demands of the role, and certain individual factors have been found to play a role in determining well-being. Coping styles are important in managing stressors, with avoidant coping generally being associated with negative outcomes (Willems et al., 2020). Other factors such as demographic characteristics, role-specific attributes, or lived experience of trauma have also been investigated. In Chinese crisis supporters, role experience was negatively correlated with burnout and secondary traumatic stress (Zhang et al., 2021). In an Australian sample, younger crisis supporters reported higher functional impairment and psychological distress (Kitchingman et al., 2017), and having a personal history of trauma was associated with increased distress from telephone-based trauma work (Dunkley & Whelan, 2006). Further research is needed to assist with identifying risk and protective factors affecting the mental well-being of crisis supporters.

Crisis support work has also evolved in recent years, to adapt to new technologies and working structures. Since the COVID-19 pandemic, crisis supporters have adapted to working remotely rather than from a helpline office, which remains an ongoing practice (Lifeline, 2022). Only one study has investigated the effects of this change, finding some evidence that feelings of isolation from remote work were linked with intention to leave (Willems et al. 2021). Moreover, despite a rapid increase in the provision of crisis support over chat, no research has been conducted investigating the differential impact of chat- and telephone-based modalities on crisis supporter well-being.

Positively, despite the stressors associated with crisis support work, many supporters can feel fulfilled by their role (Willems et al., 2020), which is integral as a significant number of them are unpaid volunteers (WHO, 2013). This fulfillment can manifest as compassion satisfaction, defined as the pleasure derived from helping others (Bride et al., 2007). In crisis supporters, compassion satisfaction is bolstered by perceived social support from other volunteers (Donnellan et al., 2023) and from friends and family (Spafford et al., 2023). As such, maintaining a supportive culture at crisis helplines may be an important factor in promoting compassion satisfaction and reducing turnover.

This study seeks to address the pressing need for high-quality research in the field of crisis supporter well-being. In crisis supporters, the study aims to:

1. identify role-related stressors;
2. investigate levels of mental well-being (including compassion fatigue, compassion satisfaction, psychological distress) and substance use; and
3. identify individual-level characteristics associated with mental well-being.

## Methods

### Procedure and Participants

An online survey was conducted using the platform Qualtrics between March and July 2023. Eligible participants were Australians aged 18 years or older and currently/recently (in the past 3 months) in a crisis supporter role. Participants were recruited through: (1) emails and newsletters distributed within four of the largest Australian crisis helpline providers and (2) social media advertising, which provided crisis supporters from other Australian crisis helplines the opportunity to participate in the study. The research team collaborated with representatives from each of the four organizations, who reviewed the survey and provided recommendations.

### Measures

The online survey included questions pertaining to participant characteristics, stressors, mental well-being, substance use, and coping styles.

#### Participant Characteristics

Participants were asked about their demographic information, specifically their age, gender, and rurality. They also provided information regarding characteristics related to their crisis supporter role, including their monthly shift hours, experience, the primary modality they provided support (telephone or chat-based), whether they were a volunteer or paid staff member, and the proportion of hours worked remotely. Additionally, participants were asked to respond to a question asking if they had a lived experience of suicide.

#### Role-Related Stressors

**Figure 1:**
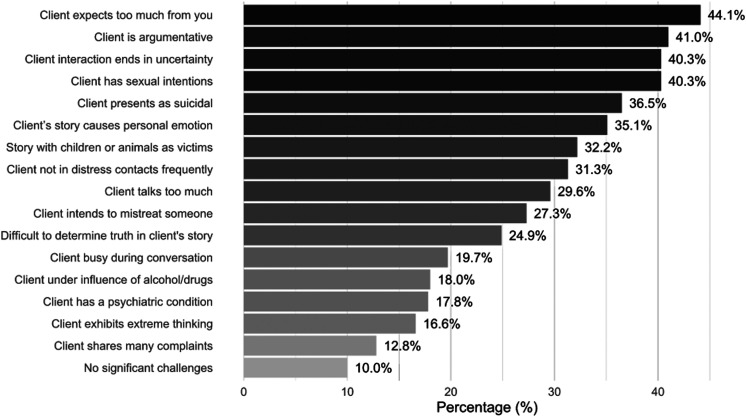
Proportion of the sample endorsing role-related stressors.

Willems et al.’s (2021) questionnaire of work-related demands was used to identify stressors. Two items were added as they were highlighted as important by Australian crisis support organizations (relating to personal triggers and uncertainty; see Figure 1). Participants were asked to select all stressors that they felt had negatively affected their mental health or well-being.

#### Compassion Fatigue and Compassion Satisfaction

The Professional Quality of Life-21 Scale (Heritage et al., 2018) was used to measure compassion fatigue and compassion satisfaction. Participants responded to 21 items on a 5-point scale (1 = *never*, 5 = *very often*). Scores were calculated for two subscales, classifying participants into low (<16 for females, <15 for males), moderate (16–24 for females, 15–24 for males), or high (≥25) levels of compassion fatigue and low (<21), moderate (21–29), or high (≥ 30) levels of compassion satisfaction. Both subscales demonstrated excellent internal consistency in the current study: compassion satisfaction (α = .92) and compassion fatigue (α = .90).

#### Psychological Distress

The Kessler Psychological Distress Scale (K10; Kessler et al., 2002) was used to measure nonspecific psychological distress in the preceding 30 days. Total scores were categorized as follows: low (10–15), moderate (16–21), high (22–29), and very high (30–50). The internal consistency of the K10 in this study was excellent (α = .92).

#### Substance Use

The Alcohol, Smoking and Substance Involvement Screening Test (ASSIST; Humeniuk et al., 2010) was used to identify risky and disordered substance use across 10 drug types. Use was categorized as low risk (≤10 for alcohol, ≤3 for other drugs), “moderate risk” (11–26 for alcohol, 4–26 for other drugs), or high risk (>27 for alcohol and other drugs). The internal consistency of the various substances was as follows: alcohol (α = .65), tobacco (α = .80), cannabis (α = .92), cocaine (α = .72), amphetamines (α = .39), inhalants (α = .95), sedatives (α = .77), hallucinogens (α = .60), opioids (α = .74), and other substances (α = .91).

#### Coping Styles

Coping styles were assessed using the 28-item self-report measure Brief COPE (Carver, 1997). Participants were asked how frequently they use different coping strategies in reference to stress from their crisis supporter role. Responses were rated on a 4-point Likert scale (1 = *I haven't been doing this at all*, 4 = *I've been doing this a lot*). The problem- (α = .89) and emotion-focused (α = .83) coping subscales displayed good internal consistency, while the avoidance coping subscale was on the margin of acceptability (α = .69).

### Ethical Approval

Ethical approval for this project was obtained from the University of Sydney Human Research Ethics Committee (protocol number: 2022/891).

### Data Analysis

A series of linear regression analyses were conducted in R (version 4.2.1; R Core Team, 2023) to examine the association between the dependent variables of compassion fatigue, compassion satisfaction, and psychological distress and 11 predetermined independent variables: gender, age, modality, shift hours, remote work proportion, employment status, experience, lived experience, and the three coping subscales. First, univariable linear regressions were conducted to establish the unadjusted associations between each independent and dependent variable. Subsequently, three separate multivariable linear regression models were run, one for each dependent variable. All independent variables were included as predictor variables in these models to account for any confounding effects. The significance level for all analyses was set at *p* < .05.

## Results

### Participant Characteristics

Participants (*n* = 422) were mostly female (82.5%), with a mean age of 46.1 (*SD* = 15.6). They conducted crisis support at 38 unique Australian crisis helplines, primarily over telephone (78.0%) and were mostly volunteers (59.2%). Participants worked a mean of 40.5 (*SD* = 45) monthly shift hours and just over half (52.6%) had been at the helpline for 2 years or less. On average, workers spent a third of their time working remotely. Over two-thirds (70.5%) had a lived experience of suicide. See Table 1 for the full breakdown of participant characteristics.

**Table 1:** Participant characteristics

Characteristic	Descriptive statistics
Gender (*n* [%])	
Female	334 (81.01)
Male	71 (17.2)
Nonbinary	7 (1.7)
Age (*M* [*SD*])	46.1 (15.6)
Geographic region (*n* [%])	
Major urban area	338 (80.9)
Regional/rural/remote	84 (19.9)
Crisis supporter role status (*n* [%])	
Paid	172 (40.8)
Volunteer	250 (59.2)
Primary modality (*n* [%])	
Telephone	329 (78.0)
Chat	93 (22.0)
Experience as crisis supporter (*n* [%])	
<1 year	101 (23.9)
1–2 years	121 (28.7)
3–5 years	92 (21.8)
≥6 years	108 (29.5)
Remote work proportion (*M*% [*SD*])	33.5 (41.7)
Hours per month (*M* [*SD*])	40.5 (45)
Lived experience of suicide ^a^ (*n* [%])	
Through suicidal ideation	169 (40.0)
Through suicide attempt	51 (12.1)
Through a loved one who has attempted/died by suicide	184 (43.6)
None	117 (27.7)
*Note*. *SD* = standard deviation. ^a^ Lived experience categories were not mutually exclusive.	

### Stressors

Figure 1 shows the proportion of participants who endorsed each role-related stressor as having had a negative impact on their mental health or well-being. The most commonly endorsed stressors *were client expects too much from you that you cannot provide as part of your role* (44.1%), *client is argumentative* (41.0%), *client has sexual intentions with the conversation* (40.3%), and client interaction ends in uncertainty (both 40.3%). Ten percent of participants indicated that they had no significant challenges in the role that negatively affected their mental health or well-being.

### Mental Well-Being and Substance Use

#### Compassion Fatigue, Satisfaction, and Psychological Distress

The mean compassion fatigue score was in the moderate range (*M* = 19.7, *SD* = 6.6). Overall, 30.6% of participants reported low levels, 49.3% moderate levels, and 20.1% high levels of compassion fatigue. For compassion satisfaction, the mean score also fell within the moderate range (*M* = 26.1, *SD* = 5.8), with 19.9% of participants exhibiting low levels, 47.6% moderate levels, and 32.5% high levels. In terms of psychological distress, the mean K10 score was classified as moderate (*M* = 18.6, *SD* = 7.5), with 44.2% scoring low, 25.5% scoring moderate, 20.2% scoring high, and 10.3% scoring very high.

#### Substance Use

The substances with the highest prevalence of lifetime use were alcohol (89.1%), cannabis (49.8%), followed by amphetamines and sedatives (both 24.0%). The least commonly used substance classes were other (2.5%), opioids (10.4%), and inhalants (11.9%). For the full ASSIST results, see Table 2.

**Table 2:** Substance use mean scores and risk categories by class of substance

Substance class	ASSIST score^a^	Abstinent	Low risk	Moderate risk	High risk
	*M* (*SD*)	*n* (%)	*n* (%)	*n* (%)	*n* (%)
Alcohol	6.9 (5.6)	44 (10.9%)	288 (71.3%)	70 (17.3%)	2 (0.5%)
Cannabis	2.1 (4.8)	203 (50.2%)	170 (42.1%)	29 (7.2%)	2 (0.5%)
Cocaine	0.9 (2.1)	324 (80.2%)	74 (18.3%)	6 (1.5%)	0 (0.0%)
Amphetamines	1.1 (2.2)	307 (76.0%)	87 (21.5%)	10 (2.5%)	0 (0.0%)
Inhalants	0.9 (3.0)	356 (88.1%)	45 (11.1%)	3 (0.7%)	0 (0.0%)
Sedatives	3.2 (4.9)	307 (76.0%)	66 (16.3%)	31 (7.7%)	0 (0.0%)
Hallucinogens	0.6 (1.5)	329 (81.4%)	71 (17.6%)	4 (1.0%)	0 (0.0%)
Opioids	1.9 (3.3)	362 (89.6%)	35 (8.7%)	7 (1.7%)	0 (0.0%)
Other	6.6 (9.4)	394 (97.5%)	5 (1.2%)	4 (1.0%)	1 (0.2%)
*Note*. *SD* = standard deviation. ^a^ASSIST score among users of each substance.					

#### Factors Associated With Mental Well-Being

The univariable regression results are presented in Electronic Supplementary Material 1 (ESM 1) and the multivariable regression results in Table 3.

**Table 3:** Multivariable regression results of associations between crisis supporter characteristics and mental well-being outcomes

Characteristic	Compassion fatigue		Compassion satisfaction		Psychological distress	
	Beta [95% CI]	*p*	Beta [95% CI]	*p*	Beta [95% CI]	*p*
Age	−0.03 [−0.07, 0.01]	.155	0.04 [0.00, 0.08]	.065	−0.11 [−0.16, −0.07]	**<.001**
Gender^a^						
Female	—		—		—	
Male	−2.12 [−3.42, −0.83]	**.001**	0.54 [−0.89, 1.97]	.458	−0.24 [−1.74, 1.27]	.756
Modality						
Telephone	—		—		—	
Chat	0.47 [−1.11, 2.05]	.557	−1.51 [−3.25, 0.22]	.087	1.91 [0.08, 3.73]	**.041**
Shift hours	0.01 [−0.01, 0.02]	.524	0.00 [−0.02, 0.02]	.901	0.00 [−0.02, 0.02]	.879
Remote work	0.02 [0.00, 0.04]	**.021**	0.00 [−0.02, 0.02]	.903	0.00 [−0.02, 0.02]	.837
Employment status				.158		
Paid	—		—		—	
Volunteer	−0.29 [−1.83, 1.25]	.711	−1.22 [−2.91, 0.48]		−0.12 [−1.91, 1.67]	.899
Experience		**.008**		.39		.702
≤1 year	—		—		—	
1–2 years	1.19 [−0.16, 2.53]	.085	0.46 [−1.02, 1.95]	.540	−0.12 [−1.68, 1.45]	.883
3–5 years	2.53 [1.05, 4.02]	**.001 **	−0.74 [−2.37, 0.89]	.375	0.70 [−1.02, 2.42]	.425
≥6 years	1.84 [0.33, 3.35]	**.017**	0.45 [−1.21, 2.10]	.597	0.61 [−1,14, 2.42]	.495
Lived experience						
Yes	—		—		—	
No	−0.91 [−1.98, 0.16]	.094	−0.94 [−2.11, 0.24]	.117	−2.15 [−3.39, −0.91]	**<.001**
Problem-focused coping	−0.14 [−0.28, −0.01]	**.042**	0.34 [0.19, 0.49]	**<.001**	−0.23 [−0.39, −0.07]	**.004**
Emotion-focused coping	0.29 [0.16, 0.43]	**<.001**	−0.18 [−0.33, −0.03]	**.017**	0.34 [0.18, 0.50]	**<.001**
Avoidant coping	1.04 [0.81, 1.28]	**<.001**	−0.74 [−1.00, −0.48]	**<.001**	1.13 [0.85, 1.40]	**<.001**
*Note.* CI = confidence Interval. ^a^Nonbinary participants were not included in analyses due to low cell counts. Bolded values indicate *p* < .05.						

#### Compassion Fatigue

Univariable analyses showed that all participant characteristics were independently associated with compassion fatigue. After including all participant characteristics in a multivariable linear regression, age, modality, shift hours, and lived experience were no longer statistically significantly associated with compassion fatigue. Gender, remote work, experience, and the coping style subscales remained statistically significant. Identifying as female, more frequent remote work, and ≥3 years of experience were associated with higher levels of compassion fatigue. Regarding coping styles, problem-focused coping had a statistically significant negative relationship with compassion fatigue. Conversely, emotion-focused coping and avoidant coping were associated with higher compassion fatigue.

#### Compassion Satisfaction

In univariable regression analyses, age, modality, remote work, emotion-focused coping, and avoidant coping were independently associated with compassion satisfaction. When all participant characteristics were included in a multivariable model, only emotion-focused coping and avoidant coping retained statistically significant associations whereby reported use of emotion-focused and avoidant coping was associated with lower compassion satisfaction scores. While not statistically significant in univariable analysis, problem-focused coping demonstrated a statistically significant positive association with compassion satisfaction in the multivariable model controlling for participant characteristics. As problem-focused coping increased, compassion satisfaction scores also increased.

#### Psychological Distress

Univariable analyses revealed that all participant characteristics, except for gender, were independently associated with distress. In the multivariable analysis, only age, modality, lived experience, and coping style retained their statistically significant associations. Specifically, younger age, providing support over chat, and having a lived experience were associated with higher distress. Problem-focused coping showed a statistically significant negative association with distress in the multivariable model, in contrast to its positive association in the univariable model. Emotion-focused coping and avoidant coping retained their statistically significant positive associations with distress in the multivariable model.

## Discussion

The present study contributes to an understudied field by investigating the mental well-being of crisis supporters. This is the first study to compare the well-being of chat-based and telephone-based crisis supporters, investigate substance use in the population, and identify stressors within the Australian helpline context. It also adds to limited research on the role of individual and role-related characteristics in determining well-being, including the effects of providing crisis support remotely.

The first aim was to identify stressors for crisis supporters. The most endorsed stressor related to clients expecting more than the crisis supporter role can provide. This is in line with qualitative research that has identified that some callers contact crisis lines for support with complex mental illness, in place of consulting healthcare professionals (Vattøe et al., 2020). This may result in crisis supporters feeling helpless to support these callers (Kitchingman et al., 2024). Other highly endorsed stressors, for example suicidal callers, or interactions ending in uncertainty have been well documented in qualitative research (Kitchingman et al., 2024; Vattøe et al., 2020; Willems et al., 2020). Such seemingly inherent stressors may be best managed by promoting healthy coping strategies among crisis supporters (see below).

Argumentative and sexually motivated callers were among the most endorsed stressors. The high accessibility of helplines can lead to misuse, with sex calls causing frustration and feelings of betrayal in crisis supporters (Kitchingman et al., 2024; Willems et al., 2020). This is a complex issue, and although helplines often have no-tolerance policies for these callers (for example: Lifeline, 2024), organizations should explore further methods of protecting their workers and volunteers from abuse.

Most crisis supporters reported low to moderate compassion fatigue. However, around one in five fell into the high compassion fatigue category. There is limited data with which to compare this finding, though it is higher than previously reported rates in Australian (Kitchingman, Caputi, et al., 2018) and American (Spafford et al., 2023) crisis supporters, where over 80% scored low levels. However, compassion fatigue was measured differently in these studies and conceptualized as a combination of burnout and secondary traumatic stress.

Rates of compassion satisfaction were high, with four in five crisis supporters reporting moderate-high levels. As many crisis supporters are volunteers, high levels of compassion satisfaction are arguably more important in this population, compared to paid caring roles where there is also a financial motivation (WHO, 2013). Feelings of reward may compensate for role-related distress and has been suggested to be a key motivator for people to stay in their role (Willems et al., 2020). The high rates of compassion satisfaction of the current sample are consistent with this theory.

Rates of high or very high levels of psychological distress were high relative to the general population (30.5% vs. 10%; Slade et al., 2011) but commensurate with those of other helping professionals (Petrie et al., 2018). Further, these levels of psychological distress are unsurprising as a large proportion of the sample had a lived experience of suicide (40% had experienced suicidal thoughts, 12.1% had attempted suicide). Given that psychological distress can also impair an individual’s crisis counseling skills (Kitchingman et al., 2017), this underscores the importance of supporting crisis workers in their role. The distress levels of crisis supporters are not only crucial for their own health but can also impact the quality of crisis helplines and have wide-reaching impacts on communities.

Levels of substance use varied greatly based on substance class. A minority of the sample was engaging in risky substance use, with alcohol, sedatives, and cannabis the highest drugs of concern. Risky substance use is often used as a coping strategy by helping professionals who encounter traumatic material as part of their job (Bonumwezi et al., 2022). This study is the first to preliminarily investigate substance use in crisis supporters, and future work should extend this research.

In the multivariable model, female gender, remote work, and experience in role were associated with increased compassion fatigue. By contrast, conducting crisis support over chat and younger age were associated with higher psychological distress. The finding that females had higher compassion fatigue is consistent with research conducted among nurses (Heritage et al., 2018). Moreover, in line with Kitchingman et al. (2017), younger crisis supporters were more at risk of experiencing psychological distress. However, this finding may be unrelated to the role, as young people have higher rates of mental ill-health in population-wide surveys (Butterworth et al., 2020).

Interestingly, crisis supporters with more experience in the role had higher compassion fatigue scores, contrasting with previous research that found that more experienced crisis supporters experienced less distress (Kitchingman et al., 2017). Compared to those with 1 year of experience or less, compassion fatigue appeared to peak for those with 3–5 years’ experience, and while still elevated for those with 6 years’ experience or more, it appeared to drop off. It may be that crisis supporters with a tenure of over 5 years report lower compassion fatigue due to survivorship bias, making a choice after a certain time as to whether they continue in the role. Taken together, organizations should take particular care in supporting crisis supporters who are female, younger, or have more experience in the role, as they may be more at risk of adverse outcomes.

On average, crisis supporters in this study worked remotely one-third of the time, with more remote work linked to higher compassion fatigue, but not to psychological distress or compassion satisfaction. These mixed findings are in accordance with Willems et al.'s (2021) study. Isolation is a significant concern of conducting crisis support from home, and the absence of face-to-face interactions can limit opportunities for supervision or informal debriefing (Button et al., 2023). While remote work adds flexibility to working arrangements, crisis helpline organizations should consider impacts on mental well-being, particularly in those who are completely remote (18.5% of this sample). Implementing strategies, such as virtual team building and peer support (Snyder et al., 2023), or providing regular opportunities for debriefing and supervision, which is already widely practiced by helplines (Furlonger & Taylor, 2013), may help to mitigate the negative effects of remote work.

Another notable finding was that chat-based crisis supporters had higher psychological distress than those providing support over telephone. Compared to telephone crisis lines, chat-based lines have been shown to have higher rates of help-seekers experiencing suicidality (Williams et al., 2021). As such, chat-based supporters may have higher exposure to traumatic material. It may also be that they have less knowledge of the outcome of interactions and may deal with more uncertainty compared to phone-based supporters. Chat-based crisis support is a rapidly growing field, and these findings emphasize a need for further research and organizational efforts to understand and address the unique challenges faced by chat-based crisis supporters.

Regarding coping styles, higher problem-focused coping was linked to positive mental well-being, replicating past findings in crisis supporter research (Kitchingman, Caputi, et al., 2018; Dunkley & Whelan, 2006). Conversely, it was found that emotion-focused coping and avoidant coping were associated with poorer mental well-being, which is consistent with research from Kitchingman, Caputi, et al. (2018) and corroborates Roche and Ogden's (2017) findings that avoidant coping was related to burnout in crisis supporters. In demanding roles like crisis support, effective coping strategies are crucial. Training at crisis support organizations should promote problem-focused coping strategies, such as reframing stressors or solution-focused journaling, and dissuade avoidant coping, such as ignoring distress resultant of the role or engaging in substance use (Carver, 1997).

### Strengths

This study had several strengths. The broad sample included crisis supporters across 38 Australian helplines, thereby increasing the generalizability of findings. The substantial sample size (*n* = 422) was obtained through a two-pronged recruitment strategy, involving social media advertising to reach crisis supporters at a variety of helplines, as well as internal communications at four major Australian crisis support organizations. The sample enabled the study to be powered to control for potential confounding factors such as participant characteristics and build a more robust picture of the correlates of well-being.

### Limitations

There are several limitations to this study that must be acknowledged. While the recruitment strategy was multifaceted, it was ultimately a convenience sample and may not be representative. For example, the sample was predominantly female, which is similar to previous studies conducted with Australian crisis supporters (Kitchingman et al., 2017) but higher than the 65% reported in workforce data (Lifeline, 2022). Additionally, although psychological distress was not included as a covariate in the compassion fatigue or satisfaction analyses, and vice versa, it is important to note that these outcomes are related and may interact with one another. Furthermore, the marginally acceptable internal consistency of the avoidant coping subscale suggests that further research may be needed to confirm relevant findings. Another limitation is the cross-sectional nature of the data. While the study was able to identify associations between variables, the results do not infer causality of relationships. Future studies of crisis supporter well-being should look to conduct longitudinal research to establish temporal antecedents of these outcomes.

### Conclusion

In summary, this study involved a robust investigation of crisis supporter mental well-being. A notable subset of crisis supporters were suffering from compassion fatigue and high psychological distress, and numerous key stressors were identified. However, high levels of compassion satisfaction were also evident. The findings of this study also speak to the importance of the relationship between coping strategies and mental well-being. Given the pivotal role crisis supporters play in suicide prevention, they should be supported in their role to ensure the resultant quality of crisis helplines. There is a critical need for research on well-being interventions for crisis supporters, as no studies have been published on this subject to date.

## Electronic Supplementary Material

The electronic supplementary material is available with the online version of the article at https://doi.org/10.1027/0227-5910/a000985

**ESM 1.** Univariable analyses.

## Notes

**Publication Ethics:** Informed consent was obtained from all participants included in the study. This study received ethical approval from the University of Sydney Human Research Ethics Committee (protocol number: 2022/891).

**Authorship:** All authors contributed to conceptualisation and writing review & editing. J.S. was responsible for writing – original draft, investigation, and formal analysis. E.D., M.D., and K.M., additionally contributed in a supervisory capacity. All authors approved the final version of the article.
