# Supplementary material for: Holding the Line – Mental Well-Being, Stressors, and Coping in Crisis Supporters
Source: Crisis. 2024 Dec 11;46(1):32–41. doi: 10.1027/0227-5910/a000985 (PMC11793082; doi:10.1027/0227-5910/a000985)
Supplement: Supplementary file 1 [file cri_46_1_32_esm1.pdf]

Electronic Supplementary Material 1 for https://doi.org/10.1027/0227-5910/a000985

Table E3. Multivariable regression results of associations between crisis supporter characteristics and mental wellbeing outcomes.

Characteristic	Compassion fatigue		Compassion satisfaction		Psychological distress	
	Beta [95%CI][1]	p-value	Beta [95%CI][1]	p-value	Beta [95%CI][1]	p-value
**Age**	-0.03 [-0.07, 0.01]	.155	0.04 [0.00, 0.08]	.065	-0.11 [-0.16, -0.07]	**<.001**
**Gender[2]**						
Female	—		—		—	
Male	-2.12 [-3.42, -0.83]	**.001**	0.54 [-0.89, 1.97]	.458	-0.24 [-1.74, 1.27]	.756
**Modality**						
Telephone	—		—		—	
Chat	0.47 [-1.11, 2.05]	.557	-1.51 [-3.25, 0.22]	.087	1.91 [0.08, 3.73]	**.041**
**Shift hours**	0.01 [-0.01, 0.02]	.524	0.00 [-0.02, 0.02]	.901	0.00 [-0.02, 0.02]	.879
**Remote work**	0.02 [0.00, 0.04]	**.021**	0.00 [-0.02, 0.02]	.903	0.00 [-0.02, 0.02]	.837
**Employment status**				.158		
Paid	—		—		—	
Volunteer	-0.29 [-1.83, 1.25]	.711	-1.22 [-2.91, 0.48]		-0.12 [-1.91, 1.67]	.899
**Experience**		**.008**		.39		.702
1 year or less	—		—		—	
1-2 years	1.19 [-0.16, 2.53]	.085	0.46 [-1.02, 1.95]	.540	-0.12 [-1.68, 1.45]	.883
3-5 years	2.53 [1.05, 4.02]	**.001**	-0.74 [-2.37, 0.89]	.375	0.70 [-1.02, 2.42]	.425
6 years or more	1.84 [0.33, 3.35]	**.017**	0.45 [-1.21, 2.10]	.597	0.61 [-1,14, 2.42]	.495
**Lived experience**						
Yes	—		—		—	
No	-0.91 [-1.98, 0.16]	.094	-0.94 [-2.11, 0.24]	.117	-2.15 [-3.39, -0.91]	**<.001**
**Problem-focused coping**	-0.14 [-0.28, -0.01]	**.042**	0.34 [0.19, 0.49]	**<.001**	-0.23 [-0.39, -0.07]	**.004**
**Emotion-focused coping**	0.29 [0.16, 0.43]	**<.001**	-0.18 [-0.33, -0.03]	**.017**	0.34 [0.18, 0.50]	**<.001**
**Avoidant coping**	1.04 [0.81, 1.28]	**<.001**	-0.74 [-1.00, -0.48]	**<.001**	1.13 [0.85, 1.40]	**<.001**

[1]CI=Confidence Interval, [2]Non-binary participants were not included in analyses due to low cell counts

© 2024 The Author(s). Distributed as a Hogrefe OpenMind article under the license CC BY 4.0 (https://creativecommons.org/licenses/by/4.0)
